# Algae and fungi move from the past to the future

**DOI:** 10.7554/eLife.49448

**Published:** 2019-07-16

**Authors:** Paola Bonfante

**Affiliations:** Department of Life Sciences and Systems BiologyUniversity of TorinoTorinoItaly

**Keywords:** *Nannochloropsis oceanica*, *Mortierella elongata*, symbiosis, isotope tracers, bioflocculation, Other

## Abstract

The ability of photosynthetic algae to enter the hyphae of a soil fungus could tell us more about the evolution of these species and their potential for applications in the production of biofuel.

**Related research article** Du ZY, Zienkiewicz K, Vande Pol N, Ostrom NE, Benning C, Bonito GM. 2019. Algal-fungal symbiosis leads to photosynthetic mycelium. *eLife* **8**:e47815. doi: 10.7554/eLife.47815

Ask a biology student to describe an association between algae and fungi and they will surely explain to you how fungal structures called hyphae can surround algal cells to form a completely new organism with its own metabolism called a lichen (Honegger, 1991; Figure 1A). Thanks to nutritional exchanges between the alga and the fungus, and adaptive mechanisms that date back some 415 million years, lichens can survive in the most extreme environments.

**Figure 1.**
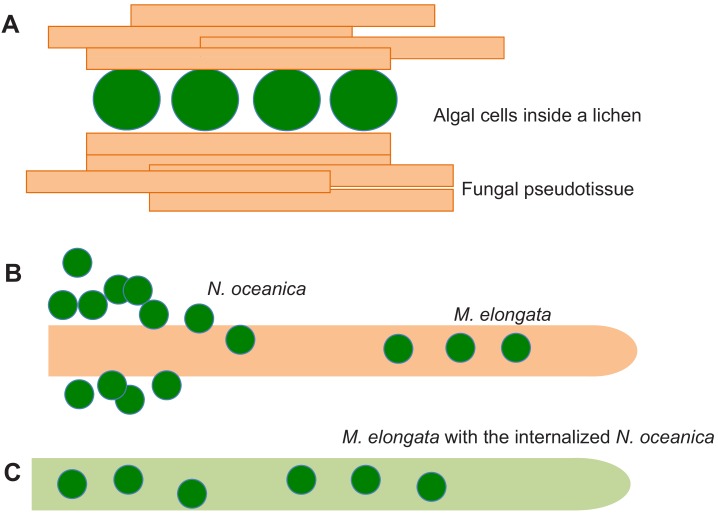
Fungal-algal symbiosis. (**A**) In lichens the algal cells (green) are surrounded by fungal hyphae (orange) to form a new organism with its own metabolism and properties. Fungal hyphae aggregate to produce fungal pseudotissues. When the alga *N. oceanica* grows in the presence of a soil fungus called *M. elongata*, the algae first aggregate and make contact with the surface of a hypha (**B**). Eventually the algae enter the hypha, which changes color to green due to the presence of the algae, which are photosynthetically active, inside it (**C**).

In all known interactions between algae and fungi, the algal cells remain outside the hyphae of the fungus. Now, in eLife, Christoph Benning, Gregory Bonito and co-workers – including Zhi-Yan Du of Michigan State University as first author – report how, under certain conditions, algal cells can enter the fungus (Du et al., 2019). The experiments were performed with *Nannochloropsis oceanica,* an algal species that lives in marine and fresh water, and *Mortierella elongata*, a fungus that lives in soil. Isotope tracer experiments revealed the exchange of nutrients, including carbon and nitrogen, between the two partners. Moreover, both remained physiologically active over two months of co-cultivation, with the algal cells continuing to grow, divide and remain photosynthetically active within the hyphae (Figure 1B,C).

Symbioses between microbes and plants or animals are often used as examples of trans-kingdom co-evolution: fossils provide direct evidence of symbiosis happening in the past, and phylogenetic analyses can reveal when the symbiotic partners appeared (Lutzoni et al., 2018; Kohler et al., 2015). For example, fossils that have been dated to the Devonian era (about 450 million years ago) reveal fungal colonization patterns that are very similar to those produced by Glomeromycotina today (Remy et al., 1994): these obligate symbionts (that is, symbionts that rely on a host to survive) enter the root cells of plants to form structures called arbuscules in a widespread form of symbiosis that is now called arbuscular mycorrhiza.

However, fossils and phylogenetics cannot tell us how the various forms of symbiosis that we see today were actually formed. Phylogenetic analyses support the idea that Glomeromycotina are members of the Mucoromycota, an early diverging fungal phylum (Spatafora et al., 2016), as is *Mortierella*. However, it is not clear if the fungi that are responsible for arbuscular mycorrhiza today evolved from saprotrophic fungi (which feed on dead or decaying matter), since there is no evidence for such fungi evolving to become obligate symbionts. By contrast, elegant experiments on the evolution of nitrogen-fixing bacteria are available: the transfer of the *sym* plasmid to a *Ralstonia* strain clearly demonstrates that a pathogen can evolve into a symbiotic bacterium (Clerissi et al., 2018).

The study of Du et al. builds on previous work which showed that the single-cell green alga *Chlamydomonas reinhardtii* and yeasts can interact under specific physiological conditions (Hom and Murray, 2014). However, the fact that *N. oceanica* and *M. elongata* mainly maintain their phenotype when they are co-cultivated is rather surprising. By contrast, the fungi in lichen aggregate and give rise to pseudotissues in which algal cells (and also bacteria) become embedded (Cardinale et al., 2008; Figure 1A).

The phenomenon observed by Du et al. started with the *N. oceanica* flocculating (that is, clumping together) around the fungus. An obvious question is: what signal causes the process of flocculation to begin? In particular, does *N. oceanica* detect and react to molecules released by *M. elongata*? Since the genomes of both partners have been sequenced, various transcriptomic and metabolomics approaches should help researchers to answer these questions.

It will also be interesting to explore if MEP α, a gene that codes for a protein that transports ammonia (NH_3_) in plants and bacteria, has a role in the exchange of nitrogen between *N. oceanica* and *M. elongata*. It is known that the MEP α gene was transferred from prokaryotes to the Leotiomyceta, which are ancestors of the fungi found in lichens, and also to the green plants (Lutzoni et al., 2018), and it is still found in all such fungi and plants. *Mortierella* and Glomeromycotina are both very rich in lipids (which contain lots of carbon), but the latter cannot synthesize lipids, relying instead on their plant host to supply them. The type of lipid exchange observed in *N. oceanica* and *M. elongata *could have applications in biotechnology. Indeed, Du et al. have shown previously that using genetic techniques to overexpress a gene called DGTT5 leads to increased lipid accumulation in *M. elongata*, which could increase the output of microalgal biofuels from this system (Du et al., 2018).

Finally, it is known that *M. elongata* is a host for various types of endobacteria (Uehling et al., 2017; Desirò et al., 2018). Since these bacteria are distinctive evolutionary markers of the ancient fungi Mucoromycota (Bonfante and Desirò, 2017), learning more about *M. elongata* may also help us to understand the biological properties of Mucoromycota that make them prone to invasion by both prokaryotes and eukaryotes.
